# A Biostimulant Seed Treatment Improved Heat Stress Tolerance During Cucumber Seed Germination by Acting on the Antioxidant System and Glyoxylate Cycle

**DOI:** 10.3389/fpls.2020.00836

**Published:** 2020-06-17

**Authors:** Cristina Campobenedetto, Eric Grange, Giuseppe Mannino, Jeroen van Arkel, Jules Beekwilder, Rumyana Karlova, Christian Garabello, Valeria Contartese, Cinzia M. Bertea

**Affiliations:** ^1^Plant Physiology Unit, Department of Life Sciences and Systems Biology, University of Turin, Turin, Italy; ^2^Green Has Italia S.p.A, Canale, Italy; ^3^BU Bioscience, Wageningen University & Research, Wageningen, Netherlands; ^4^Laboratory of Plant Physiology, Plant Sciences Group, Wageningen University & Research, Wageningen, Netherlands

**Keywords:** biostimulant, seed treatment, *Cucumis sativus*, antioxidant molecules and enzymes, isocitrate lyase, gene expression

## Abstract

Seed enhancement technologies have the potential to improve germination and seedling growth under environmental stress. The effects of KIEM^®^, an innovative biostimulant based on lignin derivatives and containing plant-derived amino acids and molybdenum, were investigated on cucumber (*Cucumis sativus* L.) seed germination. To determine the metabolic targets of this product, biometric, transcriptional and biochemical analyses were carried out on both non-treated and KIEM^®^-treated seeds incubated for 24 and 48 h under standard (28°C) and heat stress (35°C) conditions. The application of the biostimulant as a seed treatment increased the percent germination (+6.54%) and fresh biomass (+13%) at 48 h, and decreased the content of H_2_O_2_ in treated seeds at 28°C (−70%) and at 35°C (−80%). These changes in biometric and biochemical properties were accompanied by changes in expression levels of the genes coding for ROS-producing (RBOH) and scavenging (SOD, CAT, GST) enzymes and their specific activity. In general, the treatment with KIEM^®^ in heat-stress condition appeared to stimulate a higher accumulation of three scavenger gene transcripts: *CuZnSOD* (+1.78), *MnSOD* (+1.75), and *CAT* (+3.39), while the *FeSOD* isoform was dramatically downregulated (0.24). Moreover, the amount of non-protein thiols, important antioxidant molecules, was increased by the biostimulant after 48 h (+20%). Taken together these results suggest that KIEM^®^ acts through mitigation of the effects of the oxidative stress. Moreover, after 48 h, the pre-sowing treatment with KIEM^®^ increased the transcription levels (+1.5) and the activity of isocitrate lyase (+37%), a key enzyme of the glyoxylate cycle, suggesting a potential effect of this product in speeding up the germination process. Finally, the chemical characterization of KIEM^®^ identified five essential and three non-essential amino acids, and others bioactive compounds, including five organic and inorganic acids that might be potentially involved in its activity. Based on these data, insights on the potential mechanism of action of the biostimulant, suggested that there are broader applications as a product able to increase seed tolerance to different abiotic stress typical of adverse environmental conditions.

## Introduction

Plants may encounter a variety of abiotic stresses during their life cycle and these factors may have a significant impact on plant growth and final productivity. Different approaches have been employed to enhance plant stress tolerance. Some treatments can be particularly time-consuming (e.g., conventional breeding), while others are not accepted by all countries in the world (e.g., plant genetic modification). Seed priming could represent an alternative tool to prepare plants to counteract more successfully abiotic stress conditions (Filippou et al., 2013).

Recent studies suggest that different molecules have the potential to act as a biostimulant against different abiotic stresses. The application of amino acids, hormones, reactive oxygen–nitrogen–sulfur species or just water can be effective in enhancing plant tolerance to different abiotic stresses (Savvides et al., 2016). Plants can be pretreated at different developmental stages (e.g., vegetative or reproductive stage). However, in the past decades attention has been focused on seed enhancements to alleviate environmental stress on germination and early seedling growth (Taylor et al., 1998). Seed treatment technologies (Taylor, 2003) provide methods to apply synthetic or natural compounds, aimed to increase the uniformity and vigor of seedlings and to enhance the tolerance of plants to different abiotic stresses. The treatment at the seed stage has relatively low application costs, as it requires only a single treatment and often leads to a prolonged protection (Savvides et al., 2016). Biostimulant pretreatments generally cause a faster germination and a faster field emergence, which have practical agronomic implications, notably under adverse conditions (Yildirim et al., 2000).

The search for new substances able to act as biostimulant has become an important target for both the academic and seed industry. Among these new products, biostimulants could play a key role as a seed-treatment agents (Masondo et al., 2018). Modern biostimulants may be complex mixtures derived from raw materials of highly diverse origin, including waste from food and paper industries. They are considered safe for the environment and possess a broad spectrum of biological activities (Bulgari et al., 2019). In the last 25 years, plant biostimulants have received considerable attention since these innovative products offer a potentially novel approach for the modulation of physiological processes in plants to stimulate growth, to enhance stress tolerance, and to increase yield (du Jardin, 2015). For these reasons, they have found an important application in modern crop production (Yakhin et al., 2017). In general, changes in temperature significantly affect seed germination through the inhibition of radicle emergence and post-germination growth in seedlings (Probert, 2000). For this reason, the use of biostimulants to overcome heat stress became an important method to preserve the final crop production and yield (Bulgari et al., 2019).

Cucumber (*Cucumis sativus* L.) is an important vegetable crop, mainly produced in Asia and Europe, also used as model organism. Along with tomato, onion, and melon, cucumber is the most widely cultivated vegetable species in the world (Bisognin, 2002). Cucumber germination and development is negatively affected by adverse conditions, including high temperature (Kurtar, 2010; Baninasab and Ghobadi, 2011).

In this study, we evaluated the potential effects of KIEM^®^, an innovative biostimulant based on lignin derivatives (lignosulphonates) and containing plant-derived amino acids and the nutrient molybdenum (Mo) on cucumber seed germination under heat stress conditions. More than 90% of aminoacid-based biostimulants employed in agriculture is related to animal-derivative hydrolysates (Colla et al., 2015), while those of plant-origin are less common, due to their recent introduction into the biostimulant market (Colla et al., 2014). However, there are several reports that plant amino acid-based biostimulants have positive effects on seed in the early stages of germination (Yildirim et al., 2000; Ugolini et al., 2015; Amirkhani et al., 2017). Moreover, Mo is also used for seed treatments and known to be helpful during the germination process. In legumes, Mo is able to help the formation of root nodules, involved in nitrogen fixation, while in non-legume plants this micronutrient enhances the use of nitrates absorbed from the soil (Farooq et al., 2012). Application of Mo directly on seeds seems to be more effective than soil application and it could be also involved in seed protection against abiotic stress conditions, by increasing the activity of Mo-containing enzymes (Babenko et al., 2015).

In order to determine the effects and the metabolic targets of this innovative product, biometric, gene expression (qPCR) and biochemical (ROS-scavenging system) analyses were carried out on both cucumber untreated and KIEM^®^-treated seeds incubated for 24 or 48 h at 28 or 35°C (heat stress condition). Finally, in order to correlate the composition of KIEM^®^ with its possible mechanism of action, a partial chemical characterization of the amino acid fraction of this product was obtained by GC-MS analysis. All results taken together provide insights on the mechanism of action of KIEM^®^ and on its application as a seed biostimulant able to increase tolerance to heat stress and potentially to other abiotic stress typical of adverse environmental conditions. The use of KIEM^®^ as a pre-sowing agent, could be of paramount importance for reducing the number of treatments and thus the final management costs.

## Materials and Methods

### Plant Material and Biostimulant

*Cucumis sativus* L. (cucumber) seeds var. Vert Petit de Paris were purchased and certified OGM free by OLTER^®^ (Piacenza, Italy) and treated with the biostimulant KIEM^®^, developed by Green Has Italia S.p.A (Canale, Italy). This product contains 2% w/w of organic nitrogen, 2% w/w of molybdenum and 21% w/w of organic carbon. The pH (1% acq. sol. w/w) and Electrical Conductivity (acq. sol. 1 g L^–1^) were 4.00 ± 0.50 u. pH and 200 μS cm^–1^, respectively.

### Seed Treatment and Germination Parameters

Cucumber seeds were treated by following the protocol provided by Embrapa in 2005 (Henning, 2005) and currently used in Brazil for the seed treatment with different products, including phytochemicals and biostimulants (dos Santos et al., 2018). This application method allowed the use of the minimum amount of product still ensuring its homogeneous distribution on cucumber seed surface. Moreover, due to the low dosage of the product, this application method is estimated to be one of the most efficient and safe for both seeds and environment (Braccini et al., 2015; de Andrade et al., 2018; dos Santos et al., 2018). Briefly, 2 mL of KIEM^®^ solution was diluted in distilled water in order to reach the final volume of 8 mL. The KIEM^®^-diluted solution was then added drop by drop to 2.5 g of dried seeds kept in continuous shaking until the complete and visible distribution of the product on the seed surface was obtained. Following the treatment, seeds were dried at room temperature and then placed in glass Petri dishes (20 cm Ø) containing two filter papers saturated with 15 mL of distilled water. Seeds treated with the same protocol, but with distilled water instead of the biostimulant, were employed as controls. For both treatment and control, three replicates were used. Each replicate was composed by 100 seeds, placed in five different Petri dishes (20 seeds × 5 Petri Dishes). Finally, the Petri dishes were incubated in the dark at standard (28°C) or heat stress (35°C) conditions for 24 or 48 h. At 48 h, germination percentage and fresh biomass were measured in order to evaluate differences between KIEM^®^-treated and untreated seeds. Before performing the following experiments, teguments were removed from seeds and cotyledons were dry-blotted on filter paper.

### Hydrogen Peroxide Content

The hydrogen peroxide levels were detected according to Velikova et al. (2000). Powdered seeds (0.5 g) were homogenized with 5 mL of 0.1% (w/v) Trichloroacetic Acid (TCA). The samples were centrifuged at 12000 × *g* for 15 min and 0.5 mL of supernatant was added to 0.5 mL of 10 mM potassium phosphate buffer (pH 7.0) and 1 mL 1 M KI. The absorbance was read at 390 nm and the H_2_O_2_ content was determined based on a standard curve.

### Total Soluble Protein Content

The soluble protein concentration was evaluated by the method of Bradford (1976) using bovine serum albumin as a standard (Bradford, 1976).

### Non-protein Thiol Content

The assay was carried out by mixing 500 μL of crude extract prepared for enzymatic analysis (antioxidant enzyme extraction), to 100 μL of 25% (w/v) TCA. The samples were centrifuged at 12000 × *g* for 20 min at 4°C. Then, 300 μL of supernatant were added to 2.7 mL of 0.6 mM 5,5′- dithiobis (2-nitrobenzoic acid) (DTNB) prepared in 0.1 M sodium phosphate buffer (pH 8.0). The absorbance was detected at 412 nm (Jain and Bhalla-Sarin, 2001).

### Extraction and Activity of Antioxidant Enzymes

Antioxidant (ROS-producing and scavenging) enzymes were extracted and analyzed according to Contartese et al., 2016 using 0.5 g of powdered seeds (Contartese et al., 2016). All steps were carried out at 4°C. The extraction buffer used contained: 50 mM sodium phosphate, pH 7.5, 250 mM Sucrose, 1.0 mM EDTA, 10 mM KCl, 1 mM MgCl_2_, 0.5 mM phenylmethylsulfonyl fluoride (PMSF), 0.1 mM dithiothreitol (DTT), and 1% (w/v) polyvinylpolypyrrolidone (PVPP) in a 1:10 proportion (w/v). The homogenate was mixed by pipetting and then centrifuged 20 min at 25000 × *g* (4°C). The supernatants were directly used for enzymatic assays.

#### NADPH Oxidase (RBOH; EC 1.6.3.1)

The activity of RBOH was measured spectrophotometrically by reading the changes in absorbance at 530 nm (Ozawa et al., 2009). A standard assay mixture contained 40 mM NADPH, 0.02% (w/v) Triton X-100, 100 mM nitroblue tetrazolium (NBT) and buffer (20 mM Tris–chloride, pH 7.5, 3 mM MgCl_2_) to make a total volume of 1 mL in a quartz cuvette. An additional 30 μM DPI (diphenyl iodonium) was added to the reaction mixture. The specific activity was calculated using an absorption coefficient of 12.8 mM^–1^ cm^–1^.

#### Superoxide Dismutase (SOD; EC 1.15.1.1)

Superoxide dismutase activity evaluation was based on the ability of this enzyme to inhibit the reduction of nitro blue tetrazolium, thanks to the superoxide anion, generated photochemically (Krishnan et al., 2002). The reaction consisted in 1 mL containing 50 mM sodium phosphate buffer (pH 7.8), 13 mM methionine, 75 μM nitro blue tetrazolium (NBT), 2 μM riboflavin, 0.1 mM EDTA, and enzyme extract. To avoid degradation, riboflavin was added last. The samples were placed 30 cm under a light source (4000 lux) and the reaction was run for 15 min. Two blanks were prepared: one without enzyme extract, placed under the light to totally develop the reaction and, the other one, containing the enzyme extract placed in the dark to avoid the reaction. The last one was used as control. The absorbance was detected at 560 nm.

#### Catalase (CAT; EC 1.11.1.6)

Catalase activity was detected spectrophotometrically. The absorbance at 240 nm was measured for 120 s for evaluating the change due to the decreased absorption of H_2_O_2_ (ε = 39.4 mM^–1^ cm^–1^). The reaction was prepared in 1 mL final volume, containing 50 mM sodium phosphate, pH 7.0, 15 mM H_2_O_2_, and enzyme extract. The reaction was started by addition of H_2_O_2_.

#### Glutathione-S-Transferase (GST; EC 2.5.1.18)

The 1-Chloro-2,4-dinitrobenzene (CDNB) was used as reaction substrate. The enzyme activity was evaluated by monitoring the absorbance variation at 340 nm for 15 min. One mL of reaction solution contained 100 mM potassium phosphate buffer (pH 7.0), 1 mM reduced glutathione (GSH), 1 mM 1-chloro-2,4-dinitro-benzene (CDNB) (10 mM CDNB dissolved in 50% acetone stock solution), and enzyme extract. The reaction was started by adding CDNB (Jain and Bhalla-Sarin, 2001).

### Extraction and Activity of Isocitrate Lyase

All steps were carried out at 4°C. The plant material was homogenized in two volumes of extraction buffer containing 40 mM Hepes [N-(2-hydroxyethyl) piperazine-N%-(2-ethanesulfonic acid)] buffer (pH 7.0), 5 mM MgCl_2_, 1 mM EDTA, 1 mM DTT and Tween 20 (1% v/v) (Maffei et al., 1999). The homogenate was centrifuged 30 min at 15000 × *g* (4°C) and the resulting supernatants were brought to 30% saturation with solid (NH_4_)_2_SO_4_. After stirring for 2 h the solution was centrifuged at 10000 × *g* for 20 min and solid (NH_4_)_2_SO_4_ was added slowly to the supernatant to 50% saturation. After stirring for 2 h, the enzyme-enriched pellets were collected by centrifugation (10000 × *g* for 20 min), resuspended in a small volume of 40 mM Hepes (pH 7.0) and used for enzymatic assays.

*Isocitrate lyase (ICL; EC 4.1.3.1)* activity was recorded following NADH oxidation at 340 nm in the presence of an excess of lactate dehydrogenase (LDH) according to the protocol of Giachetti et al. (1987). The reaction mixture, in a final volume of 1 ml, contained: 40 mM Hepes buffer (pH 7.0), 6 mM MgCl_2_, 45 IU LDH, 0.28 mM NADH, 2 mM isocitric acid and enzyme extract. The reaction was started with isocitric acid.

### Total RNA Isolation and cDNA Synthesis

Total RNA was extracted by using the NucleoSpin^®^ RNA Plant Isolation Kit (Macherey-Nagel, Germany) according to manufacturer’s instructions. RNA concentration was measured using an UV/visible spectrophotometer Ultrospec 3000 (Pharmacia Biotech, Sweden). Total RNA quality was checked by using the RNA 6000 Nano kit and the Agilent 2100 Bioanalyzer (Agilent Technologies, United States) according to manufacturer’s instructions.

First strand cDNA synthesis was accomplished with 1 μg of total RNA and random primers using the High-Capacity cDNA Reverse Transcription Kit (Applied Biosystems, United States), according to the manufacturer’s instructions.

### Gene Expression Analysis by qPCR

All qPCR analyses were run on a Stratagene Mx3000P Real-Time System (Agilent Technologies, United States) using SYBR Green I with ROX as reference dye. The reactions were performed with 10 μL of mixture consisting of 5 μL of 2XMaxima^TM^ SYBR Green qPCR Master Mix (Thermo Fisher, United States), 0.5 μL of cDNA and 100 nM primers (Integrated DNA Technologies, United States). Thermal conditions were as follows: 10 min at 95°C, 40 cycles of 15 s at 95°C, 20 s at 57°C, and 30 s at 72°C. Fluorescence was read after each annealing and extension phase. All runs were followed by a melting curve analysis from 55 to 95°C. *Ubiquitin* (*UBI*) was used as a reference gene to normalize the results. Primers for *RBOH, CuZnSOD*, *MnSOD*, *FeSOD, CAT, GST*, *ICL*, and *UBI* used in this work are reported in Supplementary Table S1. All amplification plots were analyzed with the MX3000P^TM^ software (Agilent Technologies, United States) to obtain Ct values. The relative expression levels of each gene were estimated using the method previously described by Pfaffl (2001).

### Biostimulant Chemical Characterization

Targeted and untargeted metabolomics was performed on KIEM^®^ in order to identify polar metabolites as previously described by Lisec et al. (2006). Some adaptations were made to the protocol according to Villafort Carvalho (Villafort Carvalho et al., 2015). Briefly, polar metabolites were extracted from 50 mg of the biostimulant using methanol, followed by a 2-phase separation using chloroform. Aliquots of the polar phase were dried by vacuum centrifugation and the dried samples were derivatized online according to the protocol of Lisec et al. (2006) using a Triplus RSH autosampler system (Thermo Fischer scientific) that was coupled to the GC/MS system (Lisec et al., 2006). The derivatized samples were analyzed by gaschromatography (GC) (Thermo Trace 1300) coupled to mass spectrometry (MS) (Thermo TSQ Duo) system.

The chromatographic separation was performed using a VF−5MS capillary column [Agilent, 30 m × 0.25 mm (internal diameter) × 0.25 μm (film thickness)] including a 10-m guardian column with helium as carrier gas at a constant column flow rate of 1 ml min^–1^. The GC oven temperature was isothermal for 2 min at 70°C, followed by a 10°C min^–1^ ramp to 310°C, and then held at this temperature for 10 min. The transfer line temperature was set at 280°C. The column effluent was ionized by electron impact at 70 eV. Mass spectra were acquired using selected reaction monitoring (SRM) as scan type, with preselected SRM transitions and collision voltage. The ion source was set at a temperature of 290°C. A solvent delay of 420 s was set up. The detector voltage was set at 1500 V.

Each sample was injected in two different concentrations in order to better detect and quantify the different compounds. External calibration curves of each amino acid were used for the identification and quantification. On the other hand, other polar compounds were tentatively identified using the in-house metabolite database.

### Statistical Analysis

Data are expressed as mean values ± standard deviation (SD) of three biological replicates. Hydrogen peroxide and non-protein thiol content were expressed as nmol or μmol g^–1^ of fresh weight (FW). Enzymatic activities were expressed as nKat mg^–1^ protein, as previously described (Maffei et al., 1999). Concerning molecular data, gene expression was calculated using the method previously described by Pfaffl (2001). For all determinations, significant differences (*p* ≤ 0.05) among the samples were evaluated by performing one-way ANOVA followed by Tukey-Kramer’s s HSD test using SPSS ver. 24 software.

## Results

### Seed Germination and Fresh Biomass

The biostimulant treatment was investigated on cucumber seeds at the early germination phase under heat stress conditions, the percent germination and fresh weight were measured on control and KIEM^®^-treated seeds at 48 h after incubation at 28°C and 35°C. At 48 h, both untreated and KIEM^®^-treated seeds incubated at 28°C showed similar germination percentage (Table 1). At 24 h, seeds were imbibed and the cotyledons appeared healthy and uniform among the different replicates. Moreover, at 48 h both germination and radicle length appeared uniform. With regard to fresh weight, a significant lower weight was recorded for treated seeds compared to untreated ones (*p* ≤ 0.05). However, KIEM^®^ treatment prompted a significant increase in the germination percentage and in the fresh biomass of the germinating seeds with respect to non-treated controls (Table 1).

**TABLE 1:** Germination percentage and fresh weight at 48 h after seed incubation.

Seed treatment	Germination (%)	Fresh weight (g)
28°C Untreated	100 ± 0.58^a^	3.11 ± 0.05^a^
28°C Treated	99 ± 0.6^a^	2.85 ± 0.01^b^
35°C Untreated	92 ± 1.5^b^	1.74 ± 0.02^d^
35°C Treated	98 ± 0.6^a^	2.00 ± 0.02^c^

*Values are expressed as a mean (±SD). Different letters indicate significant differences among samples (ANOVA, Tukey’s *post hoc* test, *p* ≤ 0.05).*

### Endogenous H_2_O_2_ Content

Heat stress potentially generates a condition of oxidative stress in seeds, leading to the overproduction of ROS. For this reason, the levels of endogenous H_2_O_2_ were evaluated in untreated and KIEM^®^-treated cucumber seeds incubated in standard (28°C) and heat stress condition (35°C) for 24 and 48 h. The results are reported in Table 2. Heat stress on untreated cucumber seeds caused an increase in endogenous H_2_O_2_, both after 24 and 48 h. On the other hand, KIEM^®^-treated seeds showed strong reduction of endogenous H_2_O_2_ levels, at both incubation temperatures. Indeed, after 24 h a decrease from 1.94 ± 0.12 to 0.61 ± 0.05 nmol g^–1^ FW, and from 15.09 ± 0.57 to 3.04 ± 0.89 nmol g^–1^ FW was recorded, at 28 and 35°C, respectively. A similar trend was also observed after 48 h, in which the H_2_O_2_ content decreased from 1.94 ± 0.25 to 0.79 ± 0.04 at 28°C, and from 19.09 ± 0.15 to 1.86 ± 0.69 at 35°C. In particular, after biostimulant treatments, endogenous H_2_O_2_ decrease ranged between −70% (at 28°C) and −80% (at 35°C) after 24 h, and between −60% (at 28°C) and −90% (at 35°C) after 48 h.

**TABLE 2:** H_2_O_2_ (nmol of H_2_O_2_ g^–1^ FW) and non-protein thiol (μmol of thiols g^–1^ FW) levels at 24 and 48 h after seed incubation.

		H_2_O_2_		Non-protein thiols	
Treated	Stressed	24 h	48 h	24 h	48 h
x	x	1.95 ± 0.12^a^	1.94 ± 0.25^a^	41.0 ± 3.22^a^	38.6 ± 2.92^a^
✓	x	0.61 ± 0.06^b^	0.79 ± 0.04^b^	56.4 ± 0.58^b^	36.0 ± 2.64^ab^
x	✓	15.0 ± 0.57^c^	19.1 ± 0.15^c^	245 ± 11.2^c^	34.2 ± 2.54^b^
✓	✓	3.04 ± 0.90^d^	1.86 ± 0.70^a^	174 ± 4.84^d^	45.3 ± 3.18^c^
		*nmol of H_2_O_2_ g*^–^*^1^ FW*		μ*mol of thiols g*^–^*^1^ FW*	

*Each value is the mean ± SD of three biological replicates. For each column, different lowercase letters indicate significant differences (*p* ≤ 0.05), as measured by one-way ANOVA followed by Tukey’s *post hoc* test.*

### Non-protein Thiol Content

The levels of non-protein thiols were evaluated in untreated and KIEM^®^-treated cucumber seeds incubated in standard (28°C) and heat stress condition (35°C) for 24 and 48 h. The results are reported in Table 2. After 24 h, heat stress on untreated cucumber seeds caused a strong increase of non-protein thiol content (from 40.96 ± 2.56 to 245.26 ± 11.23 μmol g^–1^ FW). When the non-protein thiol content was measured on treated-seeds, a slight increase (+40%) was recorded with respect to untreated seeds (Table 2). On the other hand, treated seeds at 35°C displayed an opposite effect, and clearly led to a reduction in thiols (−30%) compared to the non-biostimulant control. In particular, the content was reduced from 245.23 ± 11.23 to 174.11 ± 4.84 μmol g^–1^ FW. Interestingly, an opposite trend was observed after 48 h, where an increase of the level of non-protein thiols (+35%) was observed at 35°C in KIEM^®^-treated cucumber seeds (Table 2). The delayed effect under high temperature conditions suggests a possible activation in the biostimulant-depending production of thiol molecules.

### Antioxidant Enzyme Activities and Gene Expression

To gain more insight into the seed response to heat stress and H_2_O_2_ production during the early phases of germination, the transcript levels and the activities of several ROS producing and scavenging enzymes namely, NADPH-Oxidase (RBOH), superoxide dismutase (SOD), catalase (CAT) and glutathione S transferase (GST) were evaluated.

**FIGURE 1:**
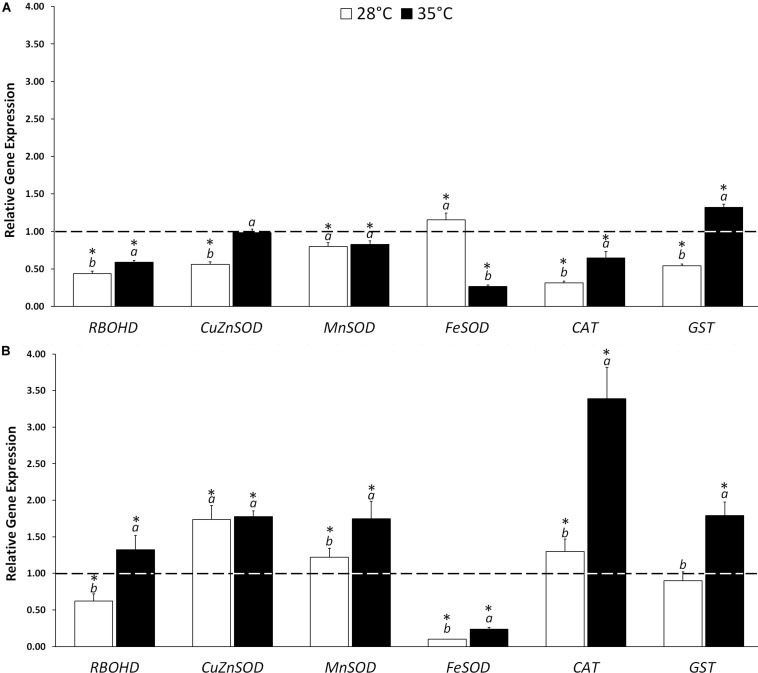
Effect of KIEM^®^ on expression levels of genes coding for ROS producing (*RBOHD*) and scavenging (*CuZnSOD*, *MnSOD*, *FeSOD*, *CAT*, and *GST*) enzymes after 24 **(A)** and 48 h **(B)** from seed incubation. Values are expressed as a relative gene expression obtained by comparing KIEM^®^-treated samples with the corresponding untreated controls (dotted line). Bars represent the mean ± SD of three biological replicates. For each bar, different lowercase letters indicate significant differences (*p* ≤ 0.05) between treatments with the biostimulant at the two different temperatures (28 and 35°C), as measured by ANOVA followed by Tukey’s *post hoc* test. Asterisks (*) indicate significant differences (*p* ≤ 0.05) between KIEM^®^-treated samples and the correspective untreated control at the same temperature condition, as measured by *t*-test.

The gene expression analysis was carried out on *RBOHD*, three *SOD* isoforms (*CuZnSOD, MnSOD, FeSOD*), *CAT* and *GST* on untreated and KIEM^®^-treated cucumber seeds incubated in standard (28°C) and in heat stress conditions (35°C) for 24 and 48 h. Figure 1 reports the data as fold-change values, as described by Pfaffl (2001).

In general, the treatment with KIEM^®^ at 28°C did not exert a strong effect on antioxidant gene expression level, after 24 and 48 h. In particular, after 24 h only *FeSOD* was slightly activated (+1.15), while the other antioxidant genes were downregulated with respect to the control (Figure 1A). A different expression profile was obtained at 48 h, in which the biostimulant treatment stimulated a higher accumulation of three scavenger gene transcripts: *CuZnSOD* (+1.74), *MnSOD* (+1.22) and *CAT* (+1.30). Interestingly, the *FeSOD* isoform, upregulated at 24 h, was dramatically downregulated at 48 h (0.11) (Figure 1B).

Also, when cucumber seeds were treated with KIEM^®^ and incubated at 35°C for 24 h, downregulation of antioxidant genes, similar to that recorded at 28°C, was observed (*MnSOD*, 0.82; *FeSOD*, 0.26, and *CAT*, 0.65) (Figure 1A). The downregulation of the genes coding for antioxidant enzymes might be correlated to the capacity of this biostimulant to slow down the consequence of heat stress. Indeed, at 24 h all the genes coding for the antioxidant enzymes in analysis, except *GST*, were downregulated. On the other hand, at 48 h, KIEM^®^ exerted a stronger effect at expression level (Figure 1B). Indeed, the treatment led to upregulation of the expression of all the antioxidant genes (*CuZnSOD*, +1.78; *MnSOD*, +1.75; *CAT*, +3.39, and *GST*, +1.7). This effect is probably due to the potential of KIEM^®^ in preparing the seedlings to be more active to counteract the effects of heat stress.

**FIGURE 2:**
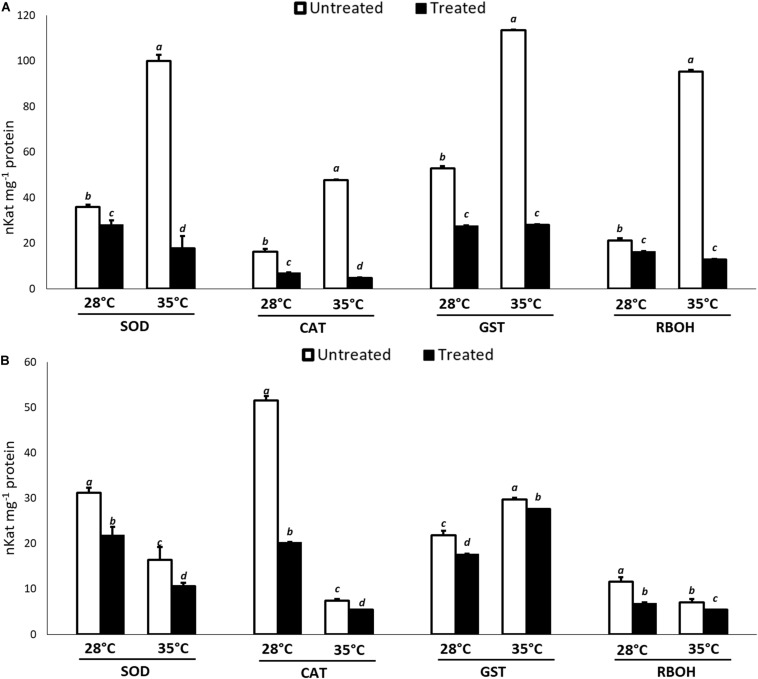
Effect of KIEM^®^ on enzymatic activities of RBOH, SOD, CAT, and GST at 24 **(A)** and 48 h **(B)** after seed incubation. Values are expressed as nKat mg^–1^ protein. Bars represent the mean ± SD of three different biological replicates. For each bar, different lowercase letters indicate significant differences (*p* ≤ 0.05), as measured by one-way ANOVA followed by Tukey’s *post hoc* test.

With regard to the enzymatic assays, in general, a lower activity of the ROS producing and scavenging enzymes compared to the controls was recorded at both temperatures and incubation times, indicated a positive action of KIEM^®^ in mitigating the effects of the oxidative stress. The data are reported in Figure 2. In particular, at 24 h (Figure 2A), the enzymatic activity profile followed the same trend as the gene expression pattern, since all enzymes showed a very low activity compared to the controls. This effect was particularly evident at 35°C, in which significant differences compared to the activity registered at 28°C were observed. After 48 h (Figure 2B) from the application of the biostimulant in heat stress conditions, a higher activity was observed for all antioxidant enzymes, compared to 24 h. The enzymatic profiles follow the observations for gene expression and data were also in agreement with the lower amount of H_2_O_2_ measured in KIEM^®^-treated seeds (Table 2).

### Isocitrate Lyase Enzymatic Activity and Gene Expression

**FIGURE 3:**
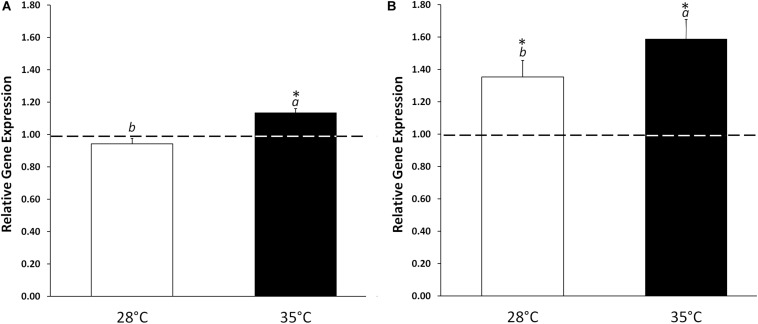
Effect of KIEM^®^ on *ICL* expression levels at 24 **(A)** and 48 h **(B)** after seed incubation. Values are expressed as a relative gene expression obtained by comparing KIEM^®^-treated samples with the corresponding untreated controls (dotted line). Bars represent the mean ± SD of three biological replicates. For each bar, different lowercase letters indicate significant differences (*p* ≤ 0.05) between treatments with the biostimulant at the two different temperatures (28 and 35°C), as measured by one-way ANOVA followed by Tukey’s *post hoc* test. Asterisks (*) indicate significant differences (*p* ≤ 0.05) between KIEM^®^-treated samples and the correspective untreated control at the same temperature condition, as measured by *t*-test.

In order to evaluate the effect of KIEM^®^ in modulating the germination process, the level of expression and the enzymatic activity of isocitrate lyase were analyzed on cucumber cotyledons treated and untreated with KIEM^®^ and incubated for 24 and 48 h at 28°C or 35°C. Isocitrate lyase, which is part of the glyoxylate pathway, is characteristic for metabolic activity of the germinating seed, catalyzing the cleavage of isocitrate to succinate and glyoxylate (Yuenyong et al., 2019). In standard conditions (28°C), KIEM^®^ did not affected the level of *ICL* expression at 24 h, but a significant (*p* ≤ 0.05) up-regulation was observed at 48 h (+1.35) when compared to control (Figure 3). The most pronounced effect was obtained when cucumber seeds were treated with KIEM^®^ at 35°C. At this temperature condition, the application of the biostimulant was able to promote *ICL* upregulation (+1.13 and +1.59, at 24 and 48 h, respectively). These data were statistically (*p* ≤ *0.*05) different not only compared to control, but to the values observed at 28°C. This suggests a positive effect of this biostimulant in enhancing the germination process, at least when judged from *ICL* expression level, at high temperature conditions.

**FIGURE 4:**
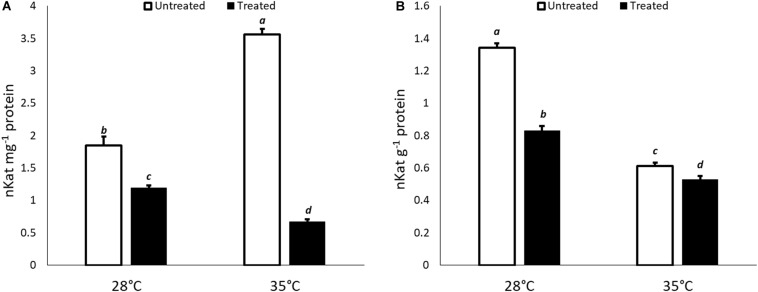
Effect of KIEM^®^ on ICL enzymatic activity at 24 **(A)** and 48 h **(B)** after seed incubation. Values are expressed as nKat mg^–1^ protein. Bars represent the mean ± SD of three different biological replicates. For each bar, different lowercase letters indicate significant differences (*p* ≤ 0.05), as measured by one-way ANOVA followed by Tukey’s *post hoc* test.

With regard to biochemical results, the changes in ICL-activity were evaluated in untreated and KIEM^®^-treated cucumber seeds incubated in standard (28°C) and heat stress condition (35°C) for 24 and 48 h. The results are reported in Figure 4. In general, a lower ICL enzymatic activity was observed in cucumber seeds treated with KIEM^®^ compared to controls at both incubation times and temperatures. However, the observed values showed a similar trend of the gene expression profile (Figure 4).

### Characterization of the Biostimulant Amino Acid Fraction

The main polar metabolites present in KIEM^®^ were analyzed through targeted and untargeted analysis using GC-MS. GC-MS analysis revealed the presence of five essential (#5, leucine; #7, isoleucine; #9, threonine; #10, methionine; and #12, phenylalanine) and three non-essential amino acids (#2, alanine; #8, serine; #14, glutamic acid) in detectable amount (Table 3). Moreover, other polar metabolites were tentatively identified comparing their retention time (RT), molecular weight (MW) and mass fragmentation (m/z) to literature data. Among them five organic and inorganic acids (#1, lactic acid; #3, sulfuric acid; #6, phosphoric acid and #15, citric acid), two sugars (#16, fructose and #18, galactose), myoinositol (#19), oxoproline (#11) and glycerol (#4) were identified. However, due to the lower and not significant amount with respect to amino acid compounds, the quantification was performed only for the amino acid fraction. GC-MS data are reported in Table 3. The most abundant amino acid found in KIEM^®^ is glutamic acid (#14) followed by methionine (#10). The sums of these two compounds counted for more of 55% of the total amino acid content.

**TABLE 3:** GC-MS analysis of the polar metabolites content in the biostimulant.

#	RT	Compound	μg per mL of KIEM^®^
1	8.21	Lactic acid	*
2	8.83	Alanine	269.2 ± 19.67^e^
3	9.79	Sulfuric acid	*
4	11.14	Glycerol	*
5	11.19	Leucine	902.3 ± 15.49^c^
6	11.19	Phosphoric acid	*
7	11.51	Isoleucine	948.4 ± 62.75^c^
8	12.32	Serine	131.2 ± 12.61^e^
9	12.65	Threonine	431.1 ± 32.44^d^
10	13.81	Methionine	1371 ± 60.28^b^
11	14.59	Oxoproline	*
12	15.77	Phenylalanine	743.1 ± 18.83^d^
13	15.84	Arabinose	*
14	17.27	Glutamic acid	2930 ± 197.9^a^
15	17.67	Citric acid	*
16	18.28	Fructose	*
17	18.42	Glucose	*
18	18.61	Galactose	*
19	20.29	Myo-inositol	*

* indicates the tentatively identified and not quantified compounds. Quantification of amino acids was performed using an external calibration curve of pure standards. Values are expressed as μg ± SD of three different replicates. Data with different lowercase letters indicate significant different values at *p* ≤ 0.05 as measured by ANOVA test followed by Tukey’s *post hoc* test. Letter “a” denotes the highest value. The symbol “*” categorizes the compounds that were tentatively identified simply on the base of their mass fragmentation and comparison with literature data.

## Discussion

The use of biostimulants to counteract the effect of abiotic stress has been documented and their capability to promote plant defenses against adverse environmental conditions were reported (Alzahrani and Rady, 2019; Rady et al., 2019). Seed treatment with biostimulants is a technology to counteract environmental stress at the time of sowing, and improving yield, all starting from seed germination (Rady et al., 2019). This is a faster method in comparison to conventional breeding or plant genetic modification and could be useful for seed treatment in countries, where high temperature at sowing could be a limiting factor (Savvides et al., 2016).

In this work, the potential effects of the biostimulant KIEM^®^ was tested on cucumber seeds germinated under standard (28°C) and heat stress (35°C) conditions by using different methodologies, such as morphological, biochemical and transcriptional (qPCR) analyses.

With regard to biometric data (Table 1), our results suggest a potential effect of KIEM^®^ in promoting germination and seedling growth under heat stress. The final germination percentage was higher in KIEM^®^-treated seeds at 48 h after incubation at 35°C, while 99% of germination percentage was observed at 28°C (Table 1). Therefore, KIEM^®^ is not harmful to seeds and has not shown phytotoxicity as biostimulants used as other studies (Yildirim et al., 2000; Masondo et al., 2018).

In seed physiology, reactive oxygen species (ROS) are usually considered as toxic molecules, whose accumulation leads to cell injury with consequent problems in seed germination and development (Jeevan Kumar et al., 2015). However, there is the increasing evidence that ROS, at low concentrations, can act as signaling molecules involved in a wide range of responses to various stimuli (Bailly, 2004; El-Maarouf-Bouteau and Bailly, 2008; Barba-Espín et al., 2012). The dual function of ROS in plants mainly relies to the cellular antioxidant machinery, which involves detoxifying enzymes (Alscher and Hess, 2017) and antioxidant compounds (Gershenzon, 1984). Such mechanisms can scavenge potentially toxic ROS, generally produced under stressful conditions, or rather tightly control ROS concentrations in order to regulate various signaling pathways. Among ROS, hydrogen peroxide plays a key role during germination process, however, high levels of H_2_O_2_ can be toxic for the seeds (Wojtyla et al., 2016). The ability of seeds to survive to this oxidative condition during germination phases is related, at least partly, to their ability to activate different detoxification systems, including both the neo-synthesis of soluble antioxidants and the activation of gene expression of enzymatic defense (Lehner et al., 2006). Our study indicates that the level of H_2_O_2_ is reduced in KIEM^®^-treated seeds (Table 2), suggesting a possible role of the biostimulant in preventing the accumulation of this reactive oxygen species. The lower amount of H_2_O_2_ observed in KIEM^®^-treated seeds can be linked to the expression level of genes coding for ROS-scavenging enzymes. The effect of KIEM^®^ seemed to be stronger at 48 h and at 35°C (Figure 1). In heat stress conditions, KIEM^®^ led to an increase in the transcription levels of all antioxidant genes, except *FeSOD*.

In addition to the scavenging enzyme machinery, plants possess a number of antioxidant molecules that are able to counteract the effects of different stress, such as non-protein thiols, the most important source of sulfur in different seeds, a fundamental element involved in metabolic pathways, nutritional quality and plant productivity.

Plants can respond to an increase in ROS production through the synthesis of soluble antioxidants (Gershenzon, 1984). Among them, non-protein thiols are considered important molecules in counteracting the effect of oxidative stress (Zagorchev et al., 2013). Thiols, such as glutathione (GSH) together with its regulation in redox signaling and defense processes, are important components for the heat stress tolerance (Szalai et al., 2009). The glutathione pool was shown to be associated with the response to heat stress of maize (Kocsy et al., 2002), *Coleus blumei* and *Fagus sylvatica* L. (Peltzer et al., 2002), *Triticum aestivum* (Nieto-Sotelo and Ho, 1986) and *Vigna radiata* (Nahar et al., 2015). Directly linked to thiols, are glutathione-S-transferases (GSTs), proteins playing important roles in enzymatic thiol-dependent ROS scavenging mechanisms (Zagorchev et al., 2013) since they catalyze the conversion of H_2_O_2_ by using glutathione or homoglutathione as substrates. In our study, the levels of non-protein thiols were higher in KIEM^®^-treated seeds compared to untreated seeds in standard conditions at 24 h and in heat stress at 48 h (Table 2), and in correlation with the expression levels of *GST* (Figure 1). The trend observed for non-protein thiols, which an initially decrease followed by an increase upon KIEM^®^ treatment, might be explained with the double role played by these molecules. These compounds are soluble antioxidants able to mitigate ROS production in several stress conditions, but they also play an important role as substrates for the synthesis of proteins and enzymes (Zagorchev et al., 2013). Moreover, GSH, the main non-protein thiol, is used as a reducing substrate in the synthesis of ascorbate (Dixit et al., 2001).

During cucumber seed germination, the glyoxylate cycle plays a key role in the mobilization of triacylglycerides located in storage tissue during post-germinative growth to effect net gluconeogenesis from the acetyl-CoA derived by β-oxidation (Lamb et al., 1978; Reynolds and Smith, 1995; Dunn et al., 2009). During early germination phases, enzymes of the glyoxylate cycle such as isocitrate lyase increase their activity during maximum fat metabolism in specialized microbodies (glyoxysomes) located in the storage tissue of germinating seeds (McLaughlin and Smith, 1994).

For these reasons, the upregulation of the gene coding for ICL is essential for the seed health status, and its down-regulation might be linked to particular stress conditions.

In general, the application of KIEM^®^ promoted a strong accumulation of *ICL* transcripts, especially at 48 h, suggesting a positive action of this biostimulant in enhancing cucumber seed germination.

In the recent years, several scientific studies reported the beneficial effects of the application of plant-derived protein hydrolysates as biostimulant in order to increase the growth, yield and fruit quality of agricultural crops (Shafeek et al., 2015). Since the beneficial properties of biostimulants were largely linked to their content of amino acids and other polar metabolites (Nardi et al., 2016), investigation about the chemical profile is actually essential to elucidate the possible mechanism of action of these products. The chemical profile of these formulations depend clearly on the raw material used for their manufacture processes, and the use of different raw materials determines changes both in metabolite profile and in plant physiological activity. With regard to seed germination, Amirkhani and co-workers showed that broccoli seeds coated by plant protein lysates enhanced seedling shoot and root growth compared to uncoated seeds (Amirkhani et al., 2016).

Despite the high content of methionine and glutamic acid in the amino acid fraction of this biostimulant, we cannot exclude that the displayed effects both on the balance of oxidative status, and on the expression of genes coding for antioxidant enzymes and isocitrate lyase might also depend on other compounds (i.e., lignin derivatives) present in the formulation of KIEM^®^, which were, however, more difficult to analyze. Probably, the effects discussed in this paper were the consequences of a synergic action of the different and several metabolites.

## Conclusion

Plant-based biostimulants are an excellent choice for a more sustainable agriculture. In this work, we showed that KIEM^®^, an innovative biostimulant, was able to increase the percent germination and restore the oxidative balance in cucumber seeds under heat stress conditions. The balancing effect is displayed not only through the reduction of endogenous H_2_O_2_ but also through the activation of antioxidant defenses. Indeed, the pre-sowing treatment with KIEM^®^ is able to restore the capacity of synthesizing the soluble antioxidants and modulate the expression of genes coding for antioxidant enzymes. Moreover, our study provided also the experimental evidence that this biostimulant is able to regulate positively the *ICL* expression, a gene coding for a key enzyme involved in the germination process. Finally, comparing the effects displayed after 24 and 48 h, it is interesting to note that the most significant protective effects occurred after 48 h from the application of the biostimulant in heat stress condition. Probably, the effects of the biostimulant were the consequence of a synergic action of the different and several metabolites in the formula. This new product may improve tolerance to heat and crop productivity by triggering different responses, along with the advantage of reducing the number of treatments and thus the final management costs.

## Data Availability Statement

All datasets generated for this study are included in the article/Supplementary Material.

## Author Contributions

CB, VC, and CG conceived and designed the experiments. VC and CG provided free of charge the biostimulant object of this study. CC, EG, JB, RK, and JA performed the experiments. GM, CC, and EG analyzed the data. GM, CC, EG, and CB wrote many parts of the manuscript. CB, CG, and VC revised the manuscript for important intellectual content. All authors read and approved the final manuscript.

## Conflict of Interest

VC and CG were employed by Green Has Italia S.p.A. CC was a Ph.D. student of the Ph.D. Program in Pharmaceutical and Biomolecular Sciences of the University of Turin, and in apprenticeship in Green Has Italia S.p.A. The remaining authors declare that the research was conducted in the absence of any commercial or financial relationships that could be construed as a potential conflict of interest.
