# Supplementary material for: A Biostimulant Seed Treatment Improved Heat Stress Tolerance During Cucumber Seed Germination by Acting on the Antioxidant System and Glyoxylate Cycle
Source: Front Plant Sci. 2020 Jun 17;11:836. doi: 10.3389/fpls.2020.00836 (PMC7311796; doi:10.3389/fpls.2020.00836)

Effect of heat-stress (35 °C) on expression levels of genes coding for ROS producing (*RBOHD*) and scavenging (*CuZnSOD*, *MnSOD*, *FeSOD*, *CAT* and *GST*) enzymes after 24 (**A**) and 48h (**B**) from seed incubation. Values are expressed as a relative gene expression obtained by comparing untreated seeds incubated in standard condition (28 °C) with untreated seeds incubated in heat-stress condition (35 °C). Bars represent the mean ± SD of three biological replicates. For each bar, different lowercase letters indicate significant differences ($p \leq 0.05$), as measured by one-way ANOVA followed by Tukey's post-hoc test.

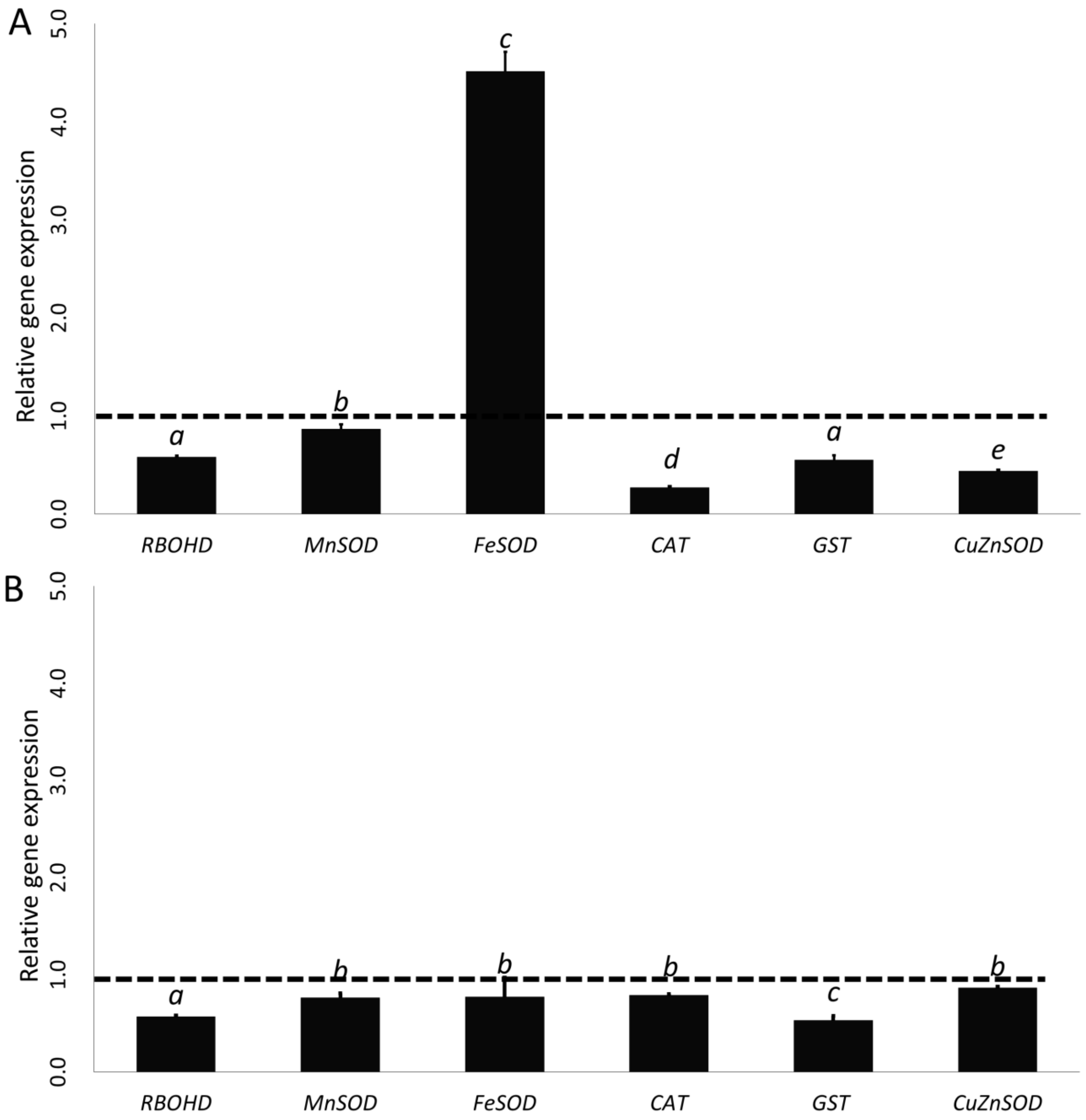

Supplement: Supplementary file 1 [file Data_Sheet_1.PDF]
