# Supplementary material for: A Biostimulant Seed Treatment Improved Heat Stress Tolerance During Cucumber Seed Germination by Acting on the Antioxidant System and Glyoxylate Cycle
Source: Front Plant Sci. 2020 Jun 17;11:836. doi: 10.3389/fpls.2020.00836 (PMC7311796; doi:10.3389/fpls.2020.00836)

Effect of heat-stress (35 °C) on expression levels of genes coding for ICL after 24 (**A**) and 48h (**B**) from seed incubation. Values are expressed as a relative gene expression obtained by comparing untreated seeds incubated in standard condition (28 °C) with untreated seeds incubated in heat-stress condition (35 °C). Bars represent the mean ± SD of three biological replicates.

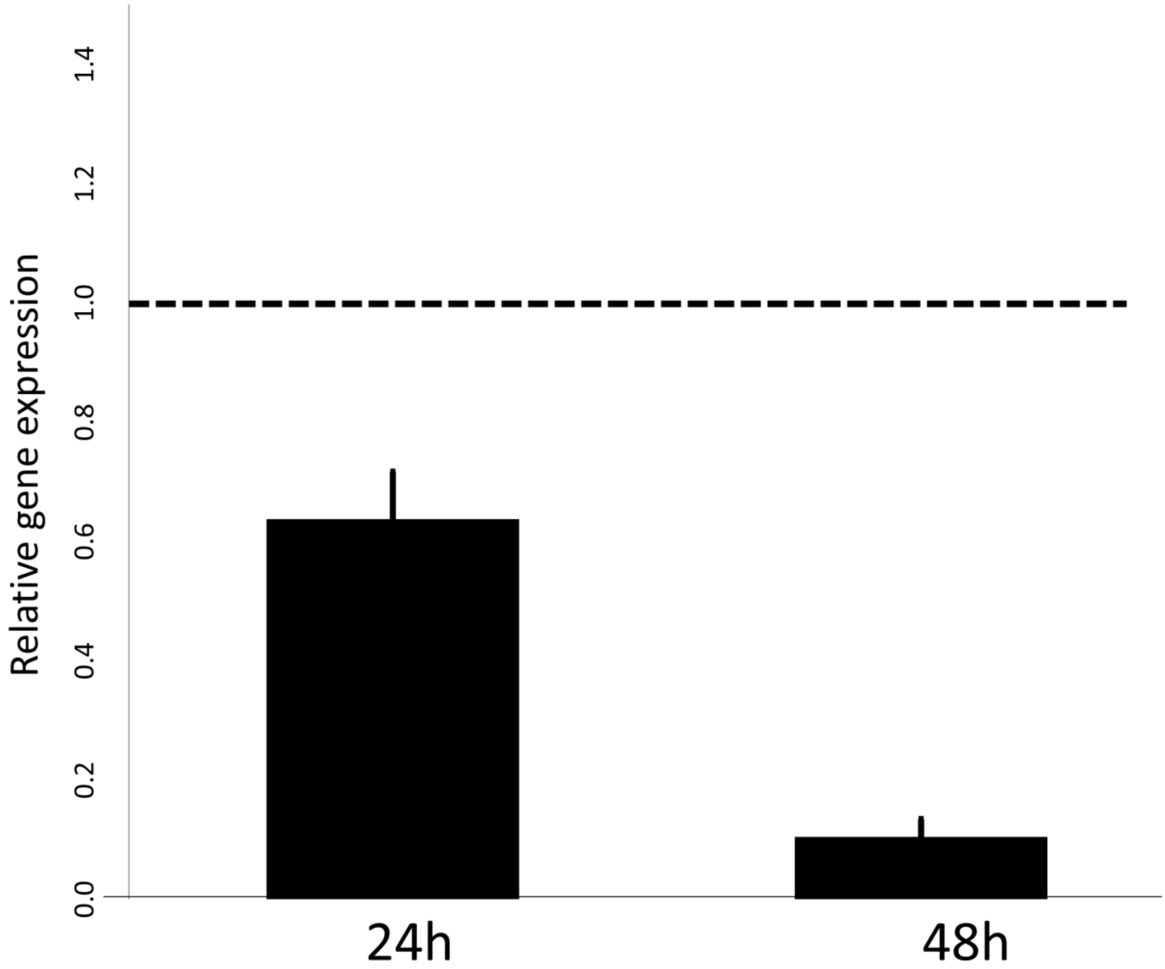

Supplement: Supplementary file 2 [file Data_Sheet_2.PDF]
