# Supplementary material for: A Biostimulant Seed Treatment Improved Heat Stress Tolerance During Cucumber Seed Germination by Acting on the Antioxidant System and Glyoxylate Cycle
Source: Front Plant Sci. 2020 Jun 17;11:836. doi: 10.3389/fpls.2020.00836 (PMC7311796; doi:10.3389/fpls.2020.00836)
Supplement: Supplementary file 3 [file Table_1.DOCX]

**Table S1.** List of primers used for qPCR analyses. **ICL* primers were designed with Primer3 software (Koressaar and Remm, 2007).

| **Accession number** | **Gene** | **Primer pairs** | **Reference** |
| --- | --- | --- | --- |
| NM_124165.3 | ***RBOHD*** | F 5’-TCTTCTTCTTCTTCCTCCCTCAAAGCC-3’  R 5’-GAAAGTTCAGGGTCTTCAAGAGAGTTGG-3’ | Jakubowska et al., 2015 |
| XM_004146841.2 | ***FeSOD*** | F 5’-ATGAAAACATACAAAAAAGG-3’  R 5’-ATGGACTCCCAGAGAAAATC-3’ | Xian et al., 2011 |
| XM_011651083.1 | ***MnSOD*** | F 5’-CAATGGCGGAGGTCACATTA-3’  R 5’-AGAGCAAGCCACACCCATC-3’ | Gao et al., 2009 |
| NM_001280768.1 | ***CuZnSOD*** | F 5’-GACTGGGCCACATTTCAACC-3’  R 5’-GCCTTGCCATCTTCACCAA-3’ | Gao et al., 2009 |
| GU248529.1 | ***CAT2*** | F 5’-ACAATCACCACGAGGGTTTC-3’  R 5’-GACAAAGCATCCACCCATCT-3’ | Hu et al., 2016 |
| XM_011652599.1 | ***GST*** | F 5’-TTTGAGGAGGTGAAGGTAA-3’  R 5’-ACGCACAAGAAATGTAGAT-3’ | Xia et al., 2011 |
| XM_004151832.2 | ***ICL*** | F 5’-TGGCACATCAGAAATGGTCT-3’  R 5’-GGACTTGGCTACCACCACAT-3’ | ***** |
| AF104391 | ***UBI*** | F 5’-CCTTATTGACCAACCAGTAGT-3’  R 5’-GGACAATGTTGATTTCCTCG-3’ | Migocka et al., 2011 |
| AB010922 | ***ACT*** | F 5’-TGGACTCTGGTGATGGTGTTA-3’  R 5’-CAATGAGGGATGGTGGAAAA-3’ | Qi et al., 2012 |

Gao, J., Li, T., Yu, X. (2009). Gene expression and activities of SOD in cucumber seedlings were related with concentration of Mn^2+^, Cu^2+^, or Zn^2+^ under low temperature stress. *Agr Sci China* 8, 678-684

Jakubowska, D., Janicka-Russak, M., Kabala, K., Migocka, M., Reda, M. (2015). Modification of plasma membrane NADPH oxidase activity in cucumber seedling roots in response to cadmium stress. Plant Science 234, 50-59

Koressaar, T and Remm M. (2007) Enhancements and modifications of primer design program Primer3. *Bioinformatics* 23, 1289-1291

Migocka, M., Papierniak, A. (2011) Identification of suitable reference genes for studying gene expression in cucumber plants subjected to abiotic stress and growth regulators. *Mol. Breeding* 28, 343-357

Qi, X.H., Xu, X.W., Lin, X.J., Zhang, W.J., Chen, X. (2012). Identification of differentially expressed genes in cucmber (*Cucumis sativus* L.) root under waterlogging stress by digital gene expression profile. Genomics 99, 160-168

Xia, X.J., Zhou, Y.H., Ding, J., Shi, K., Asami, T., Chen, Z., Yu, J.Q. (2011). Induction of systemic stress tolerance by brassinosteroids in *Cucumis sativus*. *New Phytol*. 191, 706-720
